# Tracking developments in artificial intelligence research: constructing and applying a new search strategy

**DOI:** 10.1007/s11192-021-03868-4

**Published:** 2021-02-25

**Authors:** Na Liu, Philip Shapira, Xiaoxu Yue

**Affiliations:** 1 School of Management, Shandong Technology and Business University, Yantai, 264005 China; 2 Manchester Institute of Innovation Research, Alliance Manchester Business School, University of Manchester, Manchester, M13 9PL UK; 3 School of Public Policy, Georgia Institute of Technology, Atlanta, GA 30332-0345 USA; 4 School of Public Policy and Management, Tsinghua University, Beijing, 100084 China

**Keywords:** Emerging technology, Artificial intelligence, Bibliometric analysis, Search strategy, Research trends, O31, O32, 038

## Abstract

Artificial intelligence, as an emerging and multidisciplinary domain of research and innovation, has attracted growing attention in recent years. Delineating the domain composition of artificial intelligence is central to profiling and tracking its development and trajectories. This paper puts forward a bibliometric definition for artificial intelligence which can be readily applied, including by researchers, managers, and policy analysts. Our approach starts with benchmark records of artificial intelligence captured by using a core keyword and specialized journal search. We then extract candidate terms from high frequency keywords of benchmark records, refine keywords and complement with the subject category “artificial intelligence”. We assess our search approach by comparing it with other three recent search strategies of artificial intelligence, using a common source of articles from the Web of Science. Using this source, we then profile patterns of growth and international diffusion of scientific research in artificial intelligence in recent years, identify top research sponsors in funding artificial intelligence and demonstrate how diverse disciplines contribute to the multidisciplinary development of artificial intelligence. We conclude with implications for search strategy development and suggestions of lines for further research.

## Introduction

Artificial intelligence is considered as a cutting-edge technology that is increasingly driving developments and innovations in a wide range of scientific, technological, business, and government fields (WIPO 2019a). The domain is experiencing a worldwide surge in attention from policymakers, universities and institutes, corporations and the public.

However, what is artificial intelligence and how can it be defined for bibliometric searches? Computer scientist John McCarthy and colleagues introduced the term “artificial intelligence” in a proposal for a conference held at Dartmouth College in 1956 (McCarthy et al. 1955). He later described artificial intelligence as “the science and engineering of making intelligent machines, especially computer programs” (McCarthy 2007). Subsequently, further perspectives have been put forward on what constitutes artificial intelligence.

While so far there does not appear to be a universally accepted definition of artificial intelligence (Buiten 2019; Wang 2019), there is convergence on core attributes. In his classic text, Nilsson (1998) maintains that “artificial intelligence … is concerned with intelligent behavior in artifacts” including through the development of machines that can perceive, reason, learn, communicate and act in complex environments “as well as humans can, or possibly better.” Similarly, artificial intelligence is discussed as a branch of computer science that focuses on creating systems that perform tasks usually requiring human intelligence (Chartrand et al. 2017; Russell and Bohannon 2015) or as the endowment of machines with human-like capabilities through simulating human consciousness and thinking processes using advanced algorithms or models (Jakhar and Kaur 2020). Other scholars describe artificial intelligence as a set of technologies or applications which enable machines or computers able to mimic the cognitive functions of the human brain (Tran et al. 2019). Although there are differences in standpoints as to the specific technologies and algorithmic approaches that be encompassed within meanings of artificial intelligence, examples often highlighted include machine learning, neural networks, deep learning, support vector machines, and inductive logic programming (WIPO 2019a; Morabit et al. 2019).

Meanwhile, the burgeoning in recent years of artificial intelligence applications promises to reshape economies, employment, society and governance across the world (West and Allen 2018; Dang 2019). Far reaching developments are anticipated as artificial intelligence is applied to applications such as face recognition, computer vision, biometrics, monitoring, prediction, and decision-making and transforms fields including those of finance, medicine, e-commerce, traffic management, and public security (CBInsights 2019; Zhang et al. 2019). There are expectations that artificial intelligence will free humans from repetitive tasks, generate new insights and user engagements, and boost productivity (Davenport and Ronanki 2018; Uria-Recio 2019). However, widespread concerns have also been raised about the implications of artificial intelligence for the future of work and employment as well as for widening inequities in society, ethics and bias, threats to data security, privacy, and civil liberties (British Academy 2020; Morgan et al. 2020).

The growth of artificial intelligence has been fueled by a series of scientific and technological advances across many disciplines, such as computer science, mathematics, neurosciences, engineering and linguistics, and massive improvements in computational power that enables the compilation, analysis and sharing of large volumes of data (WIPO 2019a). Public research funding and public policies have also stimulated and shaped the progression of artificial intelligence around the world (Loucks et al. 2019). While countries typically seek to deploy artificial intelligence to promote productivity, competitiveness and economic development, other goals are also variously pursued. For example, in the United States, innovation, technological leadership and national security have been emphasized; China now seeks these objectives too, alongside the use of artificial intelligence to boost manufacturing power and promote smart cities; and Japan highlights goals to bolster an aging but smart society through artificial intelligence (Appelbaum et al. 2018; Cath et al. 2018; OECD 2019; Mashiko 2020). Notably in China but also in multiple countries elsewhere, artificial intelligence for surveillance has been fostered (Feldstein 2019; Roberts et al. 2020). At the same time, in Europe, several US states, and in other countries, guidelines and policies now aim to address the ethical, data security, and privacy risks of artificial intelligence (AI HLEG 2019; EPIC 2020). Artificial intelligence has further been spurred by a ramp-up of venture capital and start-up businesses (OECD 2018; Walch 2020) as well as by massive private R&D investments especially from large corporations in the US such as Amazon, Apple, Facebook, IBM, Microsoft, and Google and in China by Alibaba, Baidu and Tencent (Webb 2019).

In this context of the worldwide rise of artificial intelligence, increasing public and private investment, anticipations of widespread applications, national strategy development, and on-going debate about its regulation and governance, approaches that can clarify the scope of this broad field and trace its research and innovation pathways are fundamental. Insights from such research and innovation mapping and tracking are vital in informing researchers, funders, companies, policymakers and other stakeholders. However, because this field is broad, dynamic and fast-moving, there are fuzzy boundaries between legacy technologies, emerging technologies and other related technologies in the artificial intelligence field (WIPO 2019a). Artificial intelligence has a legacy in computer science stretching back over seven decades. At the same time, artificial intelligence has absorbed knowledge derived from many other fields, including probability statistics, mathematics, information engineering, linguistics, game theory and neuroscience (Jackson 2019). Artificial intelligence techniques and methods are also applied in a further wide and expanding array of fields, such as speech recognition, computer vision, robotics and operations management.

In order to delineate the scope of artificial intelligence, we construct a new search strategy for bibliometric analyses of research and innovation that is able to robustly capture the variety and spread of artificial intelligence and related concepts and procedures. Our approach aims to improve upon the limited set of bibliometric approaches published to date and avoid being either too narrow or too broad. We apply a multi-stage and hybrid approach to determine relevant terms to be included in the bibliometric definition. The process involves building on, and extending from, a core corpus of scientific publications extracted from the Web of Science (WoS). The next section of this paper details our bibliometric search strategy for artificial intelligence and the steps and procedures involved. This is followed by an assessment where we undertake a comparative analysis to investigate how our search results compare with the search approaches put forward in a set of previous studies. We then use the search definition to undertake an analysis of key global trends, including growth over the last three decades, leading publishing countries and organizations, subfields, and key funding agencies. Finally, the last section of the paper highlights conclusions, limitations and some ideas for future work.

## Construction of the bibliometric search query for artificial intelligence

Bibliometric methods that analyze publications and patents are commonly used to quantitatively profile and track the development and trajectories of science and technology, including in emerging fields (Guan and Liu 2014; Liu and Guan 2016; Shapira et al. 2017; Glänzel et al. 2019). These methods typically build on search strategies that can capture relevant publications or patents in emerging fields with high recall and precision. However, the intrinsic characteristics of emerging technology domains, including their novelty, boundary ambiguities and uncertain development trajectories, present significant definitional challenges (Rotolo et al. 2015).

Among the bibliometric search approaches that are available to address these challenges are those that involve lexical keyword-based searches, the use of target domain journals, subject-category schemes, and citation and co-citation analyses (Huang et al. 2011; Arora et al. 2013). Lexical queries, using keywords, are relatively straightforward but depend on the reliability and objectivity of the expertise involved in defining keyword sets. A variation is an evolutionary lexical query with semi-automated iteration, for example by identifying core publications in an emerging field with a simple search strategy, identifying keywords and their frequency rank, repeating the search with highly-ranked keywords until convergence and involving experts in reviewing expanded keyword groups. This method still relies on the reliability of keyword selection and expert input (Huang et al. 2011, 2015). Search approaches using specific journal titles or subject categories in bibliographic databases are easily operationalized but face limitations for emerging technologies that are distributed or expanding across multiple disciplines and subject domains with outputs appearing in a widening array of journals (Huang et al. 2011; Shapira et al. 2017; Muñoz-Écija et al. 2019). Citation or co-citation search approaches start with a core set of articles exemplifying the emerging technology, adding in papers identified through citation networks and bibliographic coupling (Zitt and Bassecoulard 2006). Citation or co-citation approaches are sensitive to the starting corpus definition, have citation time-lag limitations (an issue in a fast-emerging field), and require a high level of proprietary data access (Mogoutov and Kahane 2007).

Noting that each of these methods has advantages and disadvantages, it has been recognized that bibliometric search strategies do not necessarily have to employ only one approach. Greater attention has been focused in recent years on combining methods, particularly in developing search strategies for emerging fields (Huang et al. 2015; Shapira et al. 2017; Muñoz-Écija et al. 2019; Wang et al. 2019). We similarly adopt a hybrid approach to constructing a search strategy for emerging artificial intelligence through a systematic process that takes advantage of multiple methods. Our search approach seeks to capture not only publications clearly acknowledged as artificial intelligence but also publications that should be included in the artificial intelligence field, even though their titles, abstracts or keywords may not involve the core term “artificial intelligence”.

**Fig. 1:**
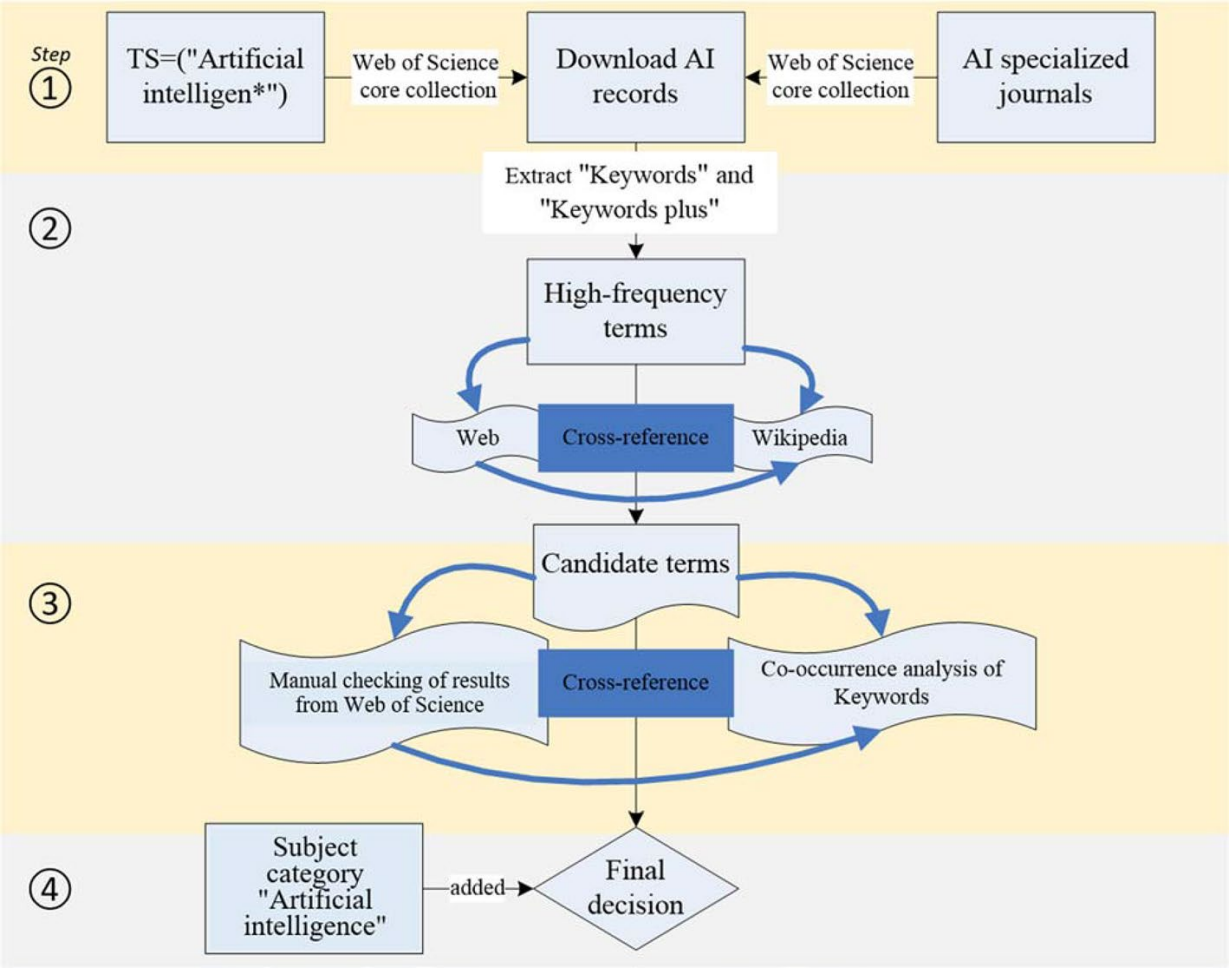
Overview of artificial intelligence search strategy

There are four key steps in the procedure we use to build a search strategy (Fig. 1). First, we generate a benchmark set of artificial intelligence publications. We use the core lexical query “artificial intelligence” as a topic search as well as a query of specialized artificial intelligence journals as a source search. Second, from these benchmark records, we extract “Author Keywords” and “Keywords Plus” and derive the frequencies of these keywords. We confirm the precise meanings of high-frequency keywords from descriptions found in online sources. This process leads to a retained list of high-frequency “candidate keywords” related to artificial intelligence. Third, to maintain balance between recall and precision, we test and refine this set of terms through co-occurrence analysis and manual checking identification. Fourth, we augment our strategy by combining the final term set with the use of a subject category search. These procedures are consecutive and are detailed in the next section.

## Artificial intelligence bibliometric search strategy

### Retrieving artificial intelligence benchmark records

Gathering benchmark records is the essential first step in our bibliometric search strategy. In the artificial intelligence field, the term “artificial intelligence” itself is extremely central. Accordingly, we use it directly as a seed search term in the “Topic” field of the WoS Science Citation Index Expanded (SCI-Expanded) and Social Sciences Citation Index (SSCI) databases. An initial search was conducted for all publication years on 21 February 2020, resulting in 24,807 records. In viewing these publications, we found that many are concerned with the application of artificial intelligence technologies in specific industrial contexts. Such papers were not relevant to our purpose of developing a conceptual search strategy. A similar observation is found in Zhou et al. (2019) in their search of “artificial intelligence” in the WoS “Title” field. To anchor our search for additional keywords germane to the core of research on artificial intelligence, we focused on the WoS subject categories of “Computer Science, Artificial Intelligence”, “Computer Science, Information Systems”, “Computer Science, Interdisciplinary Applications”, “Computer Science, Theory & Methods”, “Computer Science, Software Engineering”, “Computer Science, Hardware & Architecture”, “Computer Science, Cybernetics”, and “Robotics”. For the 9422 publication records in these eight WoS subject categories, we manually reviewed their titles and abstracts and deleted 818 records that dealt with applications. This refining process reduced the set to 8604 records.

The concept of “artificial intelligence”, as discussed in the opening parts of this paper, refers to the design of machines, programs and systems that can act with human-like reasoning and decision-making capabilities. While “artificial intelligence” is a central term, we recognized that would miss other relevant core publications if we used only this umbrella topic to identify benchmark records. To extend our core search, we also included specialized journals at the epicenter of the artificial intelligence domain. We identified 19 specialized journals that focus on artificial intelligence (Table 1). These specialized journals were chosen from the Scimago Journal Rankings for artificial intelligence (SJR 2020) and the recommended journal list of the China Computer Federation (CCF 2019). We only selected top-tier journals that focus on core artificial intelligence technologies; we eschewed journals that emphasized functional applications of artificial intelligence (for example, the journal *Artificial Intelligence in Medicine* was not selected). Of the 19 chosen top-tier journals, all are international journals; 11 are identified by both Scimago and the China Computer Federation, while the other eight are from Scimago; and all are found in the WoS and located in the subject category of “artificial intelligence”. We searched these specialized journals (all years) in the WoS on 26 February 2020. The specialized journal search resulted in a set of 32,640 records of all publication types after cleaning duplicated records.

**Table 1:** Specialized artificial intelligence journals

No.	Journal	Publisher	Year founded	Website	Publication period	Source
1	Artificial intelligence	Elsevier	1970	https://www.journals.elsevier.com/artificial-intelligence/	Monthly	Both
2	Journal of machine learning research	Microtome	2001	http://jmlr.org/	Bimonthly	Both
3	Autonomous agents and multi-agent systems	Springer	1998	https://www.springer.com/journal/10458	Bimonthly	Both
4	IEEE transactions on neural networks and learning systems	IEEE	2012	https://ieeexplore.ieee.org/xpl/RecentIssue.jsp?punumber=5962385	Monthly	Both
5	Journal of artificial intelligence research	AAAI	1993	https://www.jair.org/index.php/jair	Irregular	Both
6	Machine learning	Springer	1990	https://www.springer.com/journal/10994	Monthly	Both
7	Computational intelligence	Wiley-Blackwell	1995	https://onlinelibrary.wiley.com/journal/14678640	Quarterly	Both
8	Expert systems	Wiley-Blackwell	1994	https://onlinelibrary.wiley.com/journal/14680394	Bimonthly	Both
9	International journal of intelligent systems	Wiley	1987	https://onlinelibrary.wiley.com/journal/1098111x	Monthly	Both
10	Neurocomputing	Elsevier	1992	https://www.journals.elsevier.com/neurocomputing/	Bimonthly	Both
11	Journal of experimental and theoretical artificial intelligence	Taylor and Francis	1993	https://www.tandfonline.com/toc/teta20/current	Quarterly	Both
12	IEEE computational intelligence magazine	IEEE	2006	https://ieeexplore.ieee.org/xpl/RecentIssue.jsp?punumber=10207	Quarterly	Scimago
13	Artificial intelligence review	Springer	1988	https://www.springer.com/journal/10462	Bimonthly	Scimago
14	Autonomous robots	Springer	1996	https://www.springer.com/journal/10514	Bimonthly	Scimago
15	International journal of machine learning and cybernetics	Springer	2010	https://www.springer.com/journal/13042	Monthly	Scimago
16	ACM transactions on intelligent systems and technology	Association for Computing Machinery	2010	https://dl.acm.org/journal/tist	Bimonthly	Scimago
17	AI magazine	AAAI	1987	https://www.aaai.org/Magazine/magazine.php	Quarterly	Scimago
18	Progress in artificial intelligence	Springer	2015	https://www.springer.com/journal/13748	Quarterly	Scimago
19	Swarm intelligence	Springer	2010	https://www.springer.com/journal/11721	Quarterly	Scimago

Derived from top-tier artificial intelligence journal listings in Scimago Journal Rankings (SJR 2020) and the China Computer Federation (2019). See discussion in text. “Both” indicates nomination from both Scimago and CCF

### Adding keywords from (co-)occurrence analysis

In the second major step of our search strategy, we extracted “Author Keywords” and “Keywords Plus” from our corpus of benchmark records and counted the frequencies of these two types of keywords. We eliminated keywords that appeared fewer than three times and also some generic phases such as “system”, “design”, “information”, “complexity” and “dynamic”. Additionally, the precise meaning of each of these keywords was ascertained by checking online web sources and Wikipedia. This led to a set of high-frequency “candidate keywords” related specifically to artificial intelligence. In total, 214 candidate terms were retained, comprising 111 keywords derived from the “artificial intelligence” topic search, with a balance of 103 non-duplicated candidate terms added through the specialized journal search.

### Refining candidate keywords using (co-)occurrence analysis and hit ratio screening

Keywords that most commonly co-occurred with the central term “artificial intelligence” should themselves become part of the core lexical query. Hence, from our focal data set, we extracted benchmark records that included the keyword “artificial intelligence”. We performed a keyword co-occurrence analysis for these extracted records. This process allowed us to identify nine keywords as core lexical because they frequently co-occurred with the term “artificial intelligence”. This enabled the following “Topic Search” (TS) core lexical query for our artificial intelligence search strategy:

TS = (“Artificial Intelligen*” or “Neural Net*” or “Machine* Learning” or “Expert System$” or “Natural Language Processing” or “Deep Learning” or “Reinforcement Learning” or “Learning Algorithm$” or “*supervised Learning” or “Intelligent Agent*”).

To determine which search terms to accept among the remaining 204 candidate keywords, we introduced a simple “Hit Ratio” and performed manual checking. The search result obtained by using the ten-term core lexical query (as above) is denoted as group *A*. The search result obtained by using each of the remaining 204 candidate keywords forms group *B*. We then defined the “Hit Ratio” for each candidate keyword *C* as: HitRatio_*c*_ = (*A* ∩ *B*)/*B.* The ratio signifies how many records captured by a candidate keyword are also captured by our core lexical query. We proceed as in Huang et al. (2015) by adopting a two-step process to assess whether a candidate term should be accepted or not into the next stage of our expanded lexical query. To be specific, if HitRatio_*c*_ ≥ 70%, then we directly included the candidate keyword *C* into the expanded lexical query that is part of our final search strategy. If HitRatio_*c*_ ≤ 30%, we excluded the candidate keyword. If 30% < HitRatio_*c*_ < 70%, then a manual check was performed. For the manual check, we reviewed the search records captured by the candidate keyword *C* in the area of (*B* not (*A* ∩ *B*)). Specifically, we manually checked the abstracts of a random sample of 25 WoS records captured by the candidate keyword *C* but not captured by the core lexical query. To acquire the random sample, we sorted the records falling in the area of (*B* not (*A* ∩ *B*)) alphabetically by authors. This avoids clustering of usage changes of terms over time if sorted by publication date. We randomly selected abstracts to read and estimated how many out of each 25-record sample were related to artificial intelligence. If greater than 50% of the sample comprised publications relevant to artificial intelligence, the candidate keyword was included in our final search strategy, deeming this candidate keyword as having a low noise ratio (LR). If less than 50% of the sample were relevant artificial intelligence records, then we excluded that candidate keyword from our final search list and deemed it as having a high noise ratio (HR).

After applying the Hit Ratio procedure to the set of 204 candidate keywords, 28 candidate keywords have Hit Ratios in the range of 70.53 to 97.90% (Table 2). This indicates that more than 70% of the records searched by each keyword are also captured by our core lexical query, revealing that these keywords have a high relatedness to the field of artificial intelligence. For a further 84 candidate keywords, we find Hit Ratios between 30 and 70%. These candidate keywords were each subject to a manual check, as described above. As an example, “adaptive learning” is one of these candidate keywords. This keyword appears in 1514 published records in WoS SCI-Expanded and SSCI in the period 2010 though to 23 March 2020, of which 912 are not captured by our core lexical query. However, only 12/25 of the random record sample taken from the non-captured records were deemed to be on target and relevant for artificial intelligence research. This keyword was not added to our final search query. Another example, “multiple kernel learning” (or “multi-kernel learning” or “multikernel learning”), appears in 694 published records in the WoS databases over the same period, of which 435 are not captured by the core lexical query. In the manual check of 25 records sampled at random from the non-captured set, all were found to be related to artificial intelligence. This keyword is included in the final search set. After performing manual checks, 61 of the 84 candidate keywords were added to the final search query (Table 3).

**Table 2:** Candidate keywords directly included in the search strategy

Number	Keywords	Candidate terms	*B*	*A* ∩ *B*	Hit ratio (%)	Final decision
1	Backpropagation Learning	“Backpropagation Learning” or “Back-propagation Learning” or “Bp Learning”	381	373	97.9	Include
2	Backpropagation Algorithm	“Backpropagation Algorithm*” or “Back-propagation Algorithm*”	1348	1252	92.9	Include
3	Long Short-term Memory	“Long Short-term Memory”	2316	2111	91.2	Include
4	Pcnn	(Pcnn$ not Pcnnt) or “Pulse Coupled Neural Net*”	321	286	89.1	Include
5	Perceptron	“Perceptron$”	5836	5042	86.4	Include
6	Neuro Evolution	“Neuro-evolution” or Neuroevolution	132	114	86.4	Include
7	Liquid State Machine	“Liquid State Machine*”	47	40	85.1	Include
8	Deep Belief Net	“Deep Belief Net*”	861	723	84.0	Include
9	Radial Basis Function Network	“Radial Basis Function Net*” or Rbfnn* or “Rbf Net*”	1985	1654	83.3	Include
10	Deep Network	“Deep Net*”	1119	930	83.1	Include
11	Autoencoder	Autoencoder*	1996	1644	82.4	Include
12	Committee Machine	“Committee Machine*”	140	115	82.1	Include
13	Training Algorithm	“Training Algorithm$”	1533	1252	81.7	Include
14	Backpropagation Network	“Backpropagation Net*” or “Back-propagation Net*” or “Bp Network*”	566	456	80.6	Include
15	Q learning	“Q learning”	1218	980	80.5	Include
16	Convolutional Network	“Convolution* Net*”	1796	1443	80.4	Include
17	Actor-critic Algorithm	“Actor-critic Algorithm$”	69	55	79.7	Include
18	Feedforward Network	“Feedforward Net*” or “Feed-Forward Net*”	1168	929	79.5	Include
19	Hopfield Network	“Hopfield Net*”	198	157	79.3	Include
20	Neocognitron	Neocognitron*	46	36	78.3	Include
21	Xgboost	Xgboost*	372	288	77.4	Include
22	Boltzmann Machine	“Boltzmann Machine*”	849	655	77.2	Include
23	Activation Function	“Activation Function$”	2337	1800	77.0	Include
24	Neurodynamic Programming	“Neurodynamic Programming” or “Neuro dynamic Programming”	40	30	75.0	Include
25	Learning Model	“Learning Model*”	8007	5790	72.3	Include
26	Neurocomputing	Neurocomputing or “Neuro-Computing”	148	106	71.6	Include
27	Temporal Difference Learning	“Temporal Difference Learning”	121	86	71.1	Include
28	Echo State Network	“Echo State* Net*”	431	304	70.5	Include

Analysis of articles in SCI-E and SSCI in WoS core collection (2010-March 2020). Document type: articles; Language: English

**Table 3:** Candidate keywords subject to manual review

Number	Keywords	Candidate terms	*B*	*A* ∩ *B*	Hit ratio (%)	N	Noise Ratio	Final decision
1	Transfer Learning	“Transfer Learning”	2269	1588	70.0	21	LR	Include
2	Gradient Boosting	“Gradient Boosting”	1152	804	69.8	25	LR	Include
3	Adversarial Learning	“Adversarial Learning”	187	129	69.0	25	LR	Include
4	Feature Learning	“Feature Learning”	1574	1085	68.9	25	LR	Include
5	Heuristic Dynamic Programming	“Heuristic Dynamic Programming”	99	68	68.7	5	HR	Exclude
6	Generative Adversarial Network	“Generative Adversarial Net*”	1080	738	68.3	23	LR	Include
7	Representation Learning	“Representation Learning”	793	532	67.1	24	LR	Include
8	Multiagent Learning	“Multiagent Learning” or “Multi-agent Learning”	106	71	67.0	25	LR	Include
9	Reservoir Computing	“Reservoir Computing”	361	238	65.9	18	LR	Include
10	Co-training	“Co-training”	182	114	62.6	24	LR	Include
11	Pac Learning	“Pac Learning” or “Probabl* Approximate* Correct Learning”	64	40	62.5	25	LR	Include
12	Extreme Learning Machine	“Extreme Learning Machine*”	3842	2394	62.3	24	LR	Include
13	Instance-based Learning	“Instance-based Learning”	152	89	58.6	10	HR	Exclude
14	Recurrent Network	“Recurrent* Net*”	712	416	58.4	4	HR	Exclude
15	Competitive Learning	“Competitive Learning”	245	134	57.5	11	HR	Exclude
16	Ensemble Learning	“Ensemble Learning”	1935	1110	57.4	25	LR	Include
17	Learning Rule	“Learning Rule*”	1132	639	56.5	9	HR	Exclude
18	Propagation Algorithm	“Propagation Algorithm$”	1637	920	56.2	5	HR	Exclude
19	Machine Intelligence	“Machine* Intelligen*”	291	162	55.7	24	LR	Include
20	Neuro fuzzy	“Neuro fuzzy” or Neurofuzzy	4324	2379	55.0	25	LR	Include
21	Stochastic gradient descent	“Stochastic gradient descent”	321	585	54.9	11	HR	Exclude
22	Lazy Learning	“Lazy Learning”	64	35	54.7	25	LR	Include
23	Multiple-instance Learning	“Multi* instance Learning” or “Multiinstance Learning”	395	213	53.9	25	LR	Include
24	Multi-task Learning	“Multi* task Learning” or “Multitask Learning”	928	500	53.9	25	LR	Include
25	Computational Intelligence	“Computation* Intelligen*”	1511	813	53.8	25	LR	Include
26	Neural Model	“Neural Model*”	1411	756	53.6	25	LR	Include
27	Multi Label Learning	“Multi* Label Learning” or “Multilabel Learning”	420	225	53.6	25	LR	Include
28	Similarity Learning	“Similarity Learning”	152	78	51.3	25	LR	Include
29	Statistical Relational Learning	“Statistical Relation* Learning”	80	41	51.3	25	LR	Exclude
30	Support Vector Regression	“Support* Vector* Regression”	4655	2359	50.7	25	LR	Include
31	Manifold Regularization	“Manifold Regulari?ation”	310	157	50.7	25	LR	Include
32	Decision Forest	“Decision Forest*”	191	96	50.3	24	LR	Include
33	Generalization Error	“Generali?ation Error*”	469	232	49.5	24	LR	Include
34	Adaptive Dynamic Programming	“Adaptive Dynamic Programming” or “Approximat* Dynamic Programming”	926	457	49.4	5	HR	Exclude
35	Transductive Learning	“Transductive Learning”	122	60	49.2	25	LR	Include
36	Neurorobotics	Neurorobotic* or “Neuro-robotic*”	110	54	49.1	25	LR	Include
37	Inductive Logic Programming	“Inductive Logic Programming”	122	59	48.4	25	LR	Include
38	Natural Language Understanding	“Natural Language Understanding”	120	57	47.5	24	LR	Include
39	Adaboost	Adaboost* or “Adaptive Boosting”	1707	801	46.9	23	LR	Include
40	Incremental Learning	“Incremental Learning”	967	452	46.7	16	LR	Include
41	Random Forest	“Random Forest*”	14,190	6594	46.5	23	LR	Include
42	Cognitive Computing	“Cognitive Computing”	190	88	46.3	7	HR	Exclude
43	Metric Learning	“Metric Learning”	890	407	45.7	25	LR	Include
44	Neural Gas	“Neural Gas”	165	75	45.5	24	LR	Include
45	Grammatical Inference	“Grammatical Inference”	62	28	45.2	25	LR	Include
46	Support Vector Machine	“Support* Vector* Machine*”	34,278	15,250	44.5	20	LR	Include
47	Multi Label Classification	“Multi* Label Classification” or “Multilabel Classification”	668	297	44.5	18	LR	Include
48	Chatbot	Chatbot*	153	67	43.8	8	HR	Exclude
49	Conditional Random Field	“Conditional Random Field*”	1296	562	43.4	19	LR	Include
50	Intelligent System	“Intelligent System*”	2365	1018	43.0	11	HR	Exclude
51	Multi Class Classification	“Multi* Class Classification” or “Multiclass Classification”	1262	542	43.0	17	LR	Include
52	Mixture Of Experts	“Mixture Of Expert*”	173	74	42.8	23	LR	Include
53	Concept Drift	“Concept* Drift”	447	191	42.7	25	LR	Include
54	Genetic Programming	“Genetic Programming”	2267	957	42.2	18	LR	Include
55	String Kernel	“String Kernel*”	88	37	42.1	14	LR	Include
56	Learning To Rank	“Learning To Rank*” or “Machine-learned ranking”	395	164	41.5	25	LR	Include
57	Boosting Algorithm	“Boosting Algorithm$”	436	181	41.5	25	LR	Include
58	Robot Learning	“Robot* Learning”	200	83	41.5	21	LR	Include
59	Relevance Vector Machine	“Relevance Vector* Machine*”	550	228	41.5	25	LR	Include
60	Feature Selection	“Feature Selection”	14,472	5833	40.3	12	HR	Exclude
61	Computational Learning	“Computational Learning”	133	53	39.9	9	HR	Exclude
62	Adaptive Learning	“Adaptive Learning”	1514	602	39.8	12	HR	Exclude
63	Gradient Descent	“Gradient Descent”	3454	1327	38.4	7	HR	Exclude
64	Pattern Classification	“Pattern Classification”	2497	952	38.1	11	HR	Exclude
65	Connectionism	Connectionis*	139	53	38.1	20	LR	Include
66	Multiple Kernel Learning	“Multi* Kernel$ Learning” or “Multikernel$ Learning”	694	259	37.3	25	LR	Include
67	Graph Learning	“Graph Learning”	172	64	37.2	17	LR	Include
68	Naive Bayes Classifier	“Naive Bayes* Classifi*”	1119	412	36.8	14	LR	Include
69	Rule-based System	“Rule-based System$”	768	274	35.7	21	LR	Include
70	Classification Algorithm	“Classification Algorithm*”	5510	1960	35.6	15	LR	Include
71	Graph Kernel	“Graph* Kernel*”	198	69	34.9	21	LR	Include
72	Rule Induction	“Rule* Induction”	316	110	34.8	22	LR	Include
73	Feature Extraction	“Feature Extraction”	18,493	6368	34.4	12	HR	Exclude
74	Decision Tree	“Decision Tree*”	11,257	3848	34.2	11	HR	Exclude
75	Generative Model	“Generative Model*”	1702	569	33.4	10	HR	Exclude
76	Intelligent Control	“Intelligent Control*”	1465	487	33.2	7	HR	Exclude
77	Manifold Learning	“Manifold Learning”	1331	442	33.2	21	LR	Include
78	Structured Learning	“Structur* Learning”	1059	351	33.1	9	HR	Exclude
79	Label Propagation	“Label Propagation”	541	178	32.9	25	LR	Include
80	Hypergraph Learning	“Hypergraph* Learning”	67	22	32.8	25	LR	Include
81	Case-based Reasoning	“Case-based Reasoning”	1007	327	32.5	8	HR	Exclude
82	One Class Classifiers	“One Class Classifi*”	482	156	32.4	24	LR	Include
83	Intelligent Algorithm	“Intelligent Algorithm*”	884	285	32.2	25	LR	Include
84	Bio Inspired Computing	“Bio* Inspired Computing” or “Bioinspired Computing”	200	61	30.5	12	HR	Exclude

Analysis of articles in SCI-E and SSCI in WoS core collection (2010-March 2020). Document type: article. Language: English. *N* represents the number of records out of a 25-record random sample falling in the area of (*B* not *A* ∩ *B*) relevant artificial intelligence records. HR represents “High noise ratio”, with less than 50% of the 25-record random sample falling in the area of (*B *not *A* ∩ *B*) relevant artificial intelligence records. LR represents “Low noise ratio”, with more than 50% of the 25-record random sample falling in the area of (*B* not *A* ∩ *B*) relevant artificial intelligence records

There were 92 candidate keywords with a Hit Ratio lower than 30% (Table 4). These keywords captured records with a low degree of overlap (*A ∩ B*) with those captured by the core lexical query. These keywords were deemed as low relevance to artificial intelligence and were not included in the final search strategy. A particular example is the term “AI”, which is common abbreviation for artificial intelligence. As a candidate keyword, the Hit Ratio for “AI” is only 17.8% in terms of overlap with those records captured by the core lexical query. Based on the 30% criteria, “AI” is not included for consideration in the final search set. On investigation, we find that “AI” has multiple meanings. A search of the Acronym Finder (AF 2020), finds 164 meanings for “AI”. Of these, 50 are in science and medicine, including Adequate Intake, Adaptive Iteration, Aridity Index, Artificial Insemination, Active Ingredient, Avian Influenza, Aromatase Inhibitor and Associative Ionization. These multiple meanings of “AI” are frequent in the titles, abstracts or keywords of WoS publications. For example, “Artificial Insemination” and “AI” have a high co-occurrence (in more than 10,500 WoS publications at the time our search). This confirms that “AI” is a poor identifier for artificial intelligence publications (and we do not include it in our final search set).

**Table 4:** Candidate keywords excluded from the search strategy

Number	Keywords	Candidate terms	*B*	*A* ∩ *B*	Hit ratio (%)	Final decision
1	Cognitive Robotics	“Cognitive Robotic*”	183	54	29.5	Exclude
2	Knowledge-based System	“Knowledge-based System$”	692	202	29.2	Exclude
3	Affective Computing	“Affective Computing”	603	174	28.9	Exclude
4	Computer Vision	“Computer Vision”	11,386	3268	28.7	Exclude
5	Text Mining	“Text Mining”	5123	1467	28.6	Exclude
6	Natural Language Generation	“Natural Language Generation”	130	37	28.5	Exclude
7	Supervised Classification	“*supervised Classification”	3578	998	27.9	Exclude
8	Dictionary Learning	“Dictionary Learning”	1922	519	27.0	Exclude
9	Online Learning	“Online Learning”	4199	1129	26.9	Exclude
10	Preference Learning	“Preference Learning”	233	62	26.6	Exclude
11	Kernel Pca	“Kernel* Pca” or “Kernel* Principal Component Analys*”	750	194	25.9	Exclude
12	Data Mining	“Data Mining”	18,117	4626	25.5	Exclude
13	Anomaly Detection	“Anomaly Detection”	3525	872	24.7	Exclude
14	Artificial Immune System	“Artificial Immune System*”	689	162	23.5	Exclude
15	Kernel Method	“Kernel* Method*”	2202	493	22.4	Exclude
16	Fuzzy Logic	“Fuzzy Logic”	12,350	2762	22.4	Exclude
17	Latent Dirichlet Allocation	“Latent Dirichlet Allocation”	1084	234	21.6	Exclude
18	Gaussian Kernel	“Gaussian Kernel*”	1284	275	21.4	Exclude
19	Autonomous Learning	“Autonomous Learning”	263	56	21.3	Exclude
20	Regression Tree	“Regression Tree*”	5394	1137	21.1	Exclude
21	Pattern Recognition	“Pattern Recognition”	19,626	4136	21.1	Exclude
22	Evolutionary Computation	“Evolutionary Comput*”	2559	538	21.0	Exclude
23	Automated Planning	“Automated Planning”	248	52	21.0	Exclude
24	Firefly Algorithm	“Firefly Algorithm$”	1288	270	21.0	Exclude
25	Learning Automata	“Learning Automata” or “Learning Automaton”	523	109	20.8	Exclude
26	Bayesian Learning	“Bayes* Learning”	1117	232	20.8	Exclude
27	Topic Model	“Topic Model*”	2056	422	20.5	Exclude
28	Knowledge Representation	“Knowledge Representation”	2007	409	20.4	Exclude
29	Machine Vision	“Machine* Vision”	2666	540	20.3	Exclude
30	Granular Computing	“Granular Computing”	556	112	20.1	Exclude
31	Clonal Selection Algorithm	“Clonal Selection Algorithm$”	224	45	20.1	Exclude
32	Active Learning	“Active Learning”	3889	779	20.0	Exclude
33	Speech Recognition	“Speech Recognition”	5012	995	19.9	Exclude
34	Markov Decision Process	“Markov Decision Process*”	3032	596	19.7	Exclude
35	Probabilistic Relational Model	“Probabilistic Relational Model*”	31	6	19.4	Exclude
36	Game Tree	“Game Tree*”	88	17	19.3	Exclude
37	Big Data	“Big Data”	16,201	3027	18.7	Exclude
38	Bayesian Network	“Bayes* Net*”	6079	1103	18.1	Exclude
39	Gaussian Process	“Gaussian Process*”	6329	1139	18.0	Exclude
40	Classification Tree	“Classification Tree*”	1787	316	17.7	Exclude
41	Commonsense Reasoning	“Commonsense Reasoning”	51	9	17.7	Exclude
42	Particle Swarm Optimization	“Particle Swarm Optimi?ation”	21,909	3854	17.6	Exclude
43	Autonomous Robot	“Autonomous Robot*”	1168	201	17.2	Exclude
44	Genetic Algorithm	“Genetic Algorithm$”	49,488	8330	16.8	Exclude
45	Face Recognition	“Face Recognition”	7813	1287	16.5	Exclude
46	Probabilistic Logic	“Probabilistic Logic”	218	35	16.1	Exclude
47	Latent Semantic Analys	“Latent Semantic Analys*”	692	111	16.0	Exclude
48	Recommendation System	“Recommender System$” or “Recommendation System$”	4239	667	15.7	Exclude
49	Junction Tree	“Junction Tree*”	77	12	15.6	Exclude
50	Ambient Intelligence	“Ambient Intelligen*”	650	100	15.4	Exclude
51	Kernel Regression	“Kernel* Regression”	681	104	15.3	Exclude
52	Swarm Intelligence	“Swarm Intelligen*”	2403	364	15.2	Exclude
53	Hidden Markov Model	“Hidden Markov Model*”	6672	1008	15.1	Exclude
54	Logic Programming	“Logic Programming”	736	109	14.8	Exclude
55	Artificial Bee Colony	“Artificial Bee Colony”	2569	378	14.7	Exclude
56	Association Rule	“Association Rule*”	2377	337	14.2	Exclude
57	Autonomous Agent	“Autonomous Agent$”	923	128	13.9	Exclude
58	Ant Colony Optimization	“Ant Colony Optimi?ation”	3704	490	13.2	Exclude
59	Expectation Propagation	“Expectation Propagation”	129	17	13.2	Exclude
60	Automated Reasoning	“Automated Reasoning”	255	33	12.9	Exclude
61	Collaborative Filtering	“Collaborative Filtering”	1948	250	12.8	Exclude
62	Flower Pollination Algorithm	“Flower Pollination Algorithm$”	292	37	12.7	Exclude
63	Evolutionary Algorithm	“Evolution* Algorithm*”	13,331	1651	12.4	Exclude
64	Discriminant Analysis	“Discriminant Analys*”	18,374	2217	12.1	Exclude
65	Heuristic Search	“Heuristic Search”	1024	122	11.9	Exclude
66	Emotion Recognition	“Emotion* Recognition”	4322	508	11.8	Exclude
67	Proximal Gradient	“Proximal Gradient”	436	51	11.7	Exclude
68	Multi-agent System	“Multi* Agent System*” or “Multiagent System*”	9776	1118	11.4	Exclude
69	Bee Colony Algorithm	“Bee Colony Algorithm$”	1765	201	11.4	Exclude
70	Matrix Factorization	“Matrix Factori?ation”	6389	682	10.7	Exclude
71	Graph Mining	“Graph$ Mining” or “Graphic* Mining”	368	36	9.8	Exclude
72	Memetic Algorithm	“Memetic Algorithm$”	1147	106	9.2	Exclude
73	Multi Robot System	“Multi* Robot* System*” or “Multirobot* System*”	947	87	9.2	Exclude
74	Anytime Algorithm	“Anytime Algorithm$”	80	7	8.8	Exclude
75	Coordinate Descent	“Coordinate Descent”	1052	90	8.6	Exclude
76	Graphical Model	“Graph* Model*”	5627	468	8.3	Exclude
77	Swarm Robotics	“Swarm Robotic*”	277	23	8.3	Exclude
78	Pattern Mining	“Pattern Mining”	1115	87	7.8	Exclude
79	Structured Prediction	“Structur* Prediction”	6786	479	7.1	Exclude
80	Spatial Reasoning	“Spatial Reasoning”	358	25	7.0	Exclude
81	Cloud Computing	“Cloud Computing”	11,515	768	6.7	Exclude
82	Belief Propagation	“Belief Propagation”	1430	94	6.6	Exclude
83	Bayesian Model	“Bayes* Model*”	7859	465	5.9	Exclude
84	Em Algorithm	“Em Algorithm$”	4391	239	5.4	Exclude
85	Heuristic Algorithm	“Heuristic Algorithm$”	6998	363	5.2	Exclude
86	Clique Tree	“Clique Tree*”	41	2	4.9	Exclude
87	Bayesian Inference	“Bayes* Inference”	10,952	510	4.7	Exclude
88	Markov Chain	“Markov Chain*”	20,058	755	3.8	Exclude
89	Agent-based Model	“Agent-based Model*”	5181	165	3.2	Exclude
90	Description Logic	“Descripti* Logic”	361	11	3.1	Exclude
91	Logistic Regression	“Logistic Regression”	177,869	3620	2.0	Exclude
92	AI	“AI”	17,949	3119	17.4	Exclude

Analysis of articles in SCI-E and SSCI in WoS core collection (2010-March 2020). Document type: article. Language: English

### Final search approach

The full set of keywords for our artificial intelligence search strategy encompasses one core lexical query and two expanded lexical queries. The *core lexical query* is comprised of the ten core keywords identified at the second step of our procedure. *Expanded lexical query 1* is made up of 28 keywords whose Hit Ratio compared with the set of records generated by our core lexical query is greater than 70% (Table 2). *Expanded lexical query 2* consists of 61 manually-checked keywords with low noise ratios (Table 3). To complete the strategy, we also included the WoS subject category of “artificial intelligence” in the final search set. Scientific journals are assigned to specific categories in the WoS following consideration of their titles, scopes and citation patterns (Muñoz-Écija et al. 2019). It is recognized that subject category schemes are most helpful in delineating mature fields with relatively well-defined boundaries but insufficient for demarcating dynamic and multidisciplinary domains (Wang et al. 2019). It is thus not advisable to *exclusively* use subject categories in defining artificial intelligence. But, as a *complement* to the keyword-based approach that we have derived, the inclusion of the WoS artificial intelligence subject category adds a curated and peer-reviewed set of publications in journals that have been separately evaluated as within the field of artificial intelligence. The three lexical queries derived through the systematic procedure described in this section provide the capability to capture artificial intelligence publications across other WoS subject categories. The specialized artificial intelligence journals we identified in Table 1 are not included in the final search strategy because all of their records can be captured by the WoS subject category “artificial intelligence”. The final search approach for artificial intelligence is set out in Table 5.

**Table 5:** Final search approach for artificial intelligence

No	Search strategy	Search terms
# 1	Core lexical query	TS = (“Artificial Intelligen*” or “Neural Net*” or “Machine* Learning” or “Expert System$” or “Natural Language Processing” or “Deep Learning” or “Reinforcement Learning” or “Learning Algorithm$” or “*Supervised Learning” or “Intelligent Agent*”)
# 2	Expanded lexical query 1	TS = ((“Backpropagation Learning” or “Back-propagation Learning” or “Bp Learning”) or (“Backpropagation Algorithm*” or “Back-propagation Algorithm*”) or “Long Short-term Memory” or ((Pcnn$ not Pcnnt) or “Pulse Coupled Neural Net*”) or “Perceptron$” or (“Neuro-evolution” or Neuroevolution) or “Liquid State Machine*” or “Deep Belief Net*” or (“Radial Basis Function Net*” or Rbfnn* or “Rbf Net*”) or “Deep Net*” or Autoencoder* or “Committee Machine*” or “Training Algorithm$” or (“Backpropagation Net*” or “Back-propagation Net*” or “Bp Network*”) or “Q learning” or “Convolution* Net*” or “Actor-critic Algorithm$” or (“Feedforward Net*” or “Feed-Forward Net*”) or “Hopfield Net*” or Neocognitron* or Xgboost* or “Boltzmann Machine*” or “Activation Function$” or (“Neurodynamic Programming” or “Neuro dynamic Programming”) or “Learning Model*” or (Neurocomputing or “Neuro-Computing”) or “Temporal Difference Learning” or “Echo State* Net*”)
# 3	Expanded lexical query 2	TS = (“Transfer Learning” or “Gradient Boosting” or “Adversarial Learning” or “Feature Learning” or “Generative Adversarial Net*” or “Representation Learning” or (“Multiagent Learning” or “Multi-agent Learning”) or “Reservoir Computing” or “Co-training” or (“Pac Learning” or “Probabl* Approximate* Correct Learning”) or “Extreme Learning Machine*” or “Ensemble Learning” or “Machine* Intelligen*” or (“Neuro fuzzy” or Neurofuzzy) or “Lazy Learning” or (“Multi* instance Learning” or “Multiinstance Learning”) or (“Multi* task Learning” or “Multitask Learning”) or “Computation* Intelligen*” or “Neural Model*” or (“Multi* label Learning” or “Multilabel Learning”) or “Similarity Learning” or “Statistical Relation* Learning” or “Support* Vector* Regression” or “Manifold Regulari?ation” or “Decision Forest*” or “Generali?ation Error*” or “Transductive Learning” or (Neurorobotic* or “Neuro-robotic*”) or “Inductive Logic Programming” or “Natural Language Understanding” or (Adaboost* or “Adaptive Boosting”) or “Incremental Learning” or “Random Forest*” or “Metric Learning” or “Neural Gas” or “Grammatical Inference” or “Support* Vector* Machine*” or (“Multi* label Classification” or “Multilabel Classification”) or “Conditional Random Field*” or (“Multi* class Classification” or “Multiclass Classification”) or “Mixture Of Expert*” or “Concept* Drift” or “Genetic Programming” or “String Kernel*” or (“Learning To Rank*” or “Machine-learned Ranking”) or “Boosting Algorithm$” or “Robot* Learning” or “Relevance Vector* Machine*” or Connectionis* or (“Multi* Kernel$ Learning” or “Multikernel$ Learning”) or “Graph Learning” or “Naive bayes* Classifi*” or “Rule-based System$” or “Classification Algorithm*” or “Graph* Kernel*” or “Rule* induction” or “Manifold Learning” or “Label Propagation” or “Hypergraph* Learning” or “One class Classifi*” or “Intelligent Algorithm*”)
#4	WoS category	WC = (“Artificial Intelligence”)
#5	Total	#1 OR #2 OR #3 OR #4

## Comparative analysis of different search strategies for artificial intelligence

In the context of discussion about contrasting bibliometric search strategies and methods to define emerging fields, as highlighted earlier in this paper, it is appropriate and desirable to compare results from new approaches with those available in other studies. To undertake such benchmarking for our search approach, we undertook a comparative analysis of search strategies and results with three other recent bibliometric studies of artificial intelligence.

In the first study—an analysis of research on artificial intelligence—Gao et al. (2019) acknowledged the wide range of the artificial intelligence research domain, although they use a fairly straightforward and restricted topic search in the WoS based on TS = (“artificial intelligence”). In the second study, where artificial intelligence was examined to detect technological recombination, Zhou et al. (2019) apply a title search TI = (AI or “artificial intelligence”) in the WoS. For the time period of their search, 374 publications were found, from which 23 core papers were identified. Keywords were extracted from these articles and also combined with expert review to add expanded search terms. In the third study, presenting worldwide trends in innovation in artificial intelligence, WIPO (2019a) applied a search strategy based on patent classification codes and an extended keyword list drawing on literature review, established hierarchies, web resources, and manual checking. An artificial intelligence publication search strategy was derived from this, querying about 60 words or phrases specific to artificial intelligence concepts across all subject areas in the Scopus scientific publication database and about 35 words or phrases related to artificial intelligence applied to the Scopus subject areas of Mathematics, Computer Science, and Engineering (WIPO 2019b).

**Fig. 2:**
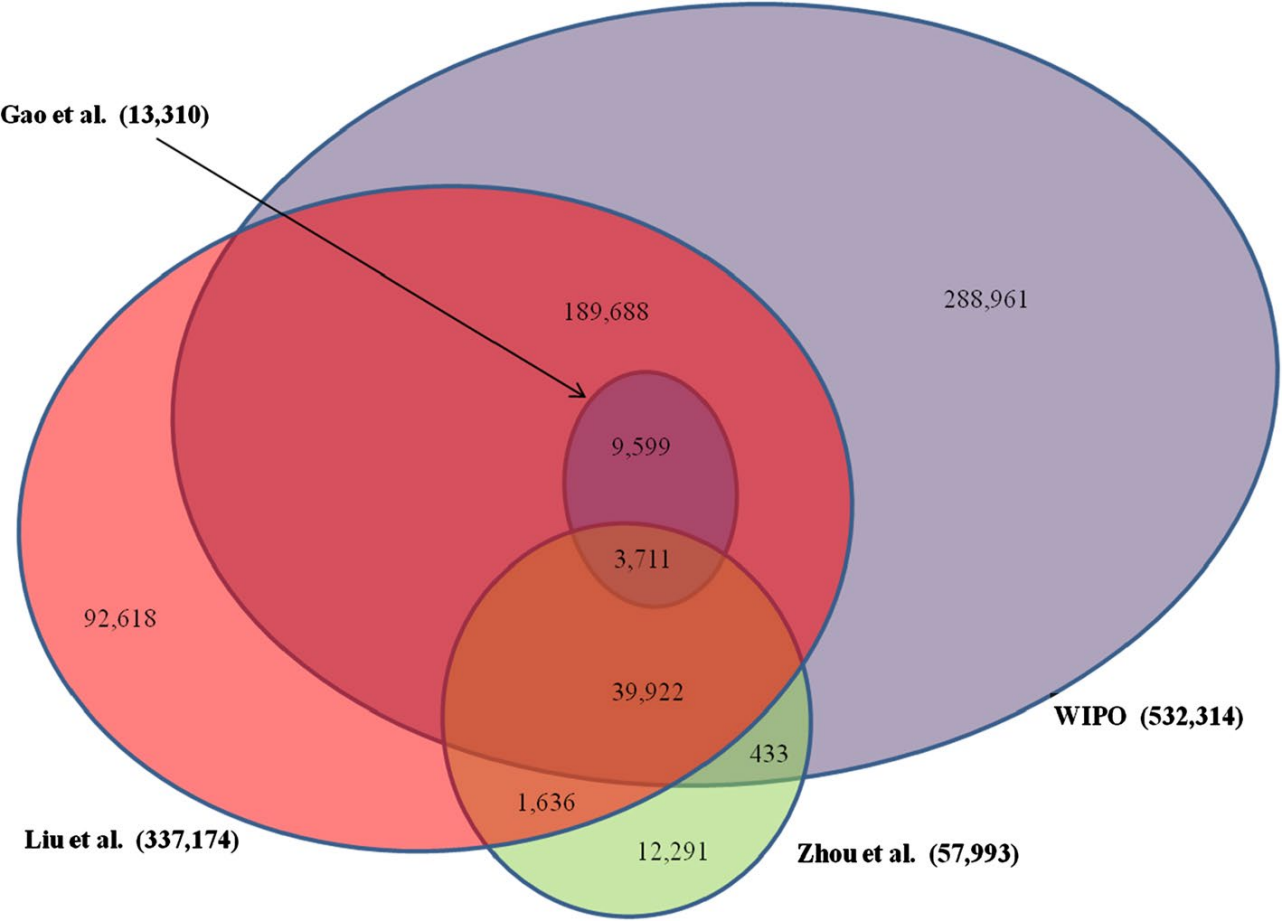
Comparison of four artificial intelligence bibliometric search strategies. *Note* Analysis of WoS (SCI-E and SSCI) artificial intelligence articles (2010–28 May 2020). See text for details including references for search strategies

We compared these three search strategies with ours. (For convenience, we refer to our approach as Liu et al.) As published, there are variations among these search strategies by bibliographic record sources, time periods, and document types analyzed. Hence, to normalize the comparison, we applied all search strategies to articles in WoS SCI-Expanded and SSCI for the time period 2010 to 28 May 2020. An analysis of the results obtained reveals overlaps as well as significant differences (Fig. 2). Gao et al.'s simple and limited approach returned 13,310 articles. The search strategy of Zhou et al. garnered 57,993 articles. But less than one third of the articles identified by Gao et al. can be captured by Zhou et al., notwithstanding that multiple additional keywords were included in the search strategy of Zhou et al. In contrast, WIPO’s broad search strategy returned the largest set, comprising 532,314 articles, covering all records captured by Gao et al. and 76% of the records captured by Zhou et al. The search strategy (Liu et al.) put forward in this paper yielded 337,174 articles, covering all the records captured by Gao et al. and just over 78% of the records captured by Zhou et al.

The WIPO search strategy has a total return that is 37% larger than our search strategy. There is a core of shared records between these two approaches: 72% of the records identified by our search strategy are also identified by WIPO, although only 46% of the records returned by WIPO are covered by our search strategy (given WIPO’s larger total return). Put another way, more than one-half (54%) of WIPO’s search result is comprised of records not included in our definition of “artificial intelligence”. We investigated the causes of this significant difference. Several generic statistical and mathematical terms such as “logistic regression”, “hidden markov model” and “fuzzy logic” are included by WIPO but excluded by us. These three terms returned 195,477 article records in the search period. The largest subject categories captured were in the fields of public health and medicine, where a manual check indicated very few papers related to artificial intelligence. About 2% of the 195,477 records were in the WoS subject category of “artificial intelligence” and just 4% in the more comprehensive WoS research area of “computer science”. Only 5334 of these records are identified in our search strategy.

Overall, the simple definition of Gao et al., with the use of just one search term “artificial intelligence”, appears to have relatively high precision but rather low recall in its limited return of article records. Zhou et al. include additional keywords, but their search also performs weakly in recall because they fail to capture artificial intelligence articles that explicitly use the term “artificial intelligence” in the “Topic” field. Conversely, WIPO’s approach has broad recall, but at the expense of precision, as a significant number of records captured are evidently extraneous to the domain of artificial intelligence. Our approach not in the arithmetic middle in this comparison of search approaches: it is in the third upper quintile of the range. While we independently include many artificial intelligence terms also identified by WIPO, our careful checking of all candidate terms means that we only include those that perform well with low noise, resulting in a search strategy that we would maintain has an appropriate balance between recall and precision.

## Trends and patterns of research in artificial intelligence

In this section, we profile and track the development and patterns of scientific research in artificial intelligence by analyzing the publication records derived from our search strategy. We investigate publication outputs and growth, citations, co-author collaborations across countries, research sponsors and scientific disciplines.

The record set used for these analyses stems from applying our search strategy (Table 5) to the WoS SCI-Expanded and SSCI databases for publication years covering the last three decades. The specific period covered is 1991 (1 January) to 2020 (24 May), an inclusive period of 29 years and 4.8 months. (In the balance of this paper, reference to 2020* denotes the period from 1 January 2020 until 24 May 2020.) After limiting our search to journal articles, excluding proceedings papers, book chapters, retracted papers, and other miscellaneous or duplicated records, our dataset of artificial intelligence scientific articles comprised 464,373 articles.

### Artificial intelligence publication outputs

**Fig. 3:**
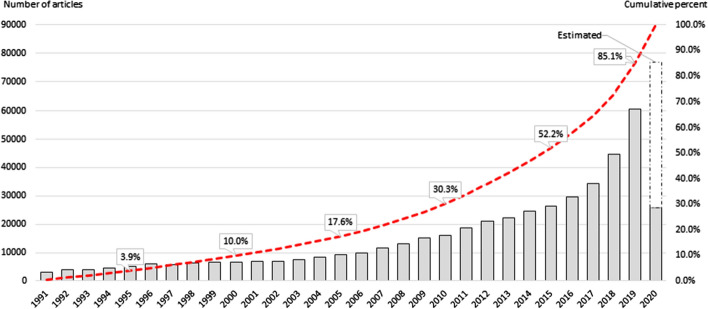
Artificial intelligence publication outputs, 1991–2020*. *Note* Analysis of WoS (SCI-E and SSCI) artificial intelligence articles published 1991–2020* (*N* = 464,373). Columns represent annual article output. Dotted line represents cumulative percent of articles. Annualized total for 2020 estimated from averaged annual growth rates for prior 3 years. Countries identified by author affiliations. 2020* = 24 May 2020

An analysis of publication trends, worldwide, for artificial intelligence articles shows continuous growth from 1991 through to 2020* (Fig. 3). An exponential growth trajectory is evident, beginning with a relatively slower growth in the first 10 years from 1991, accelerating from the mid-to-late 2000s, with a further boost in momentum from 2016. Almost half of all artificial intelligence articles produced between 1991 and 2020* were published in the most recent five years.

**Fig. 4:**
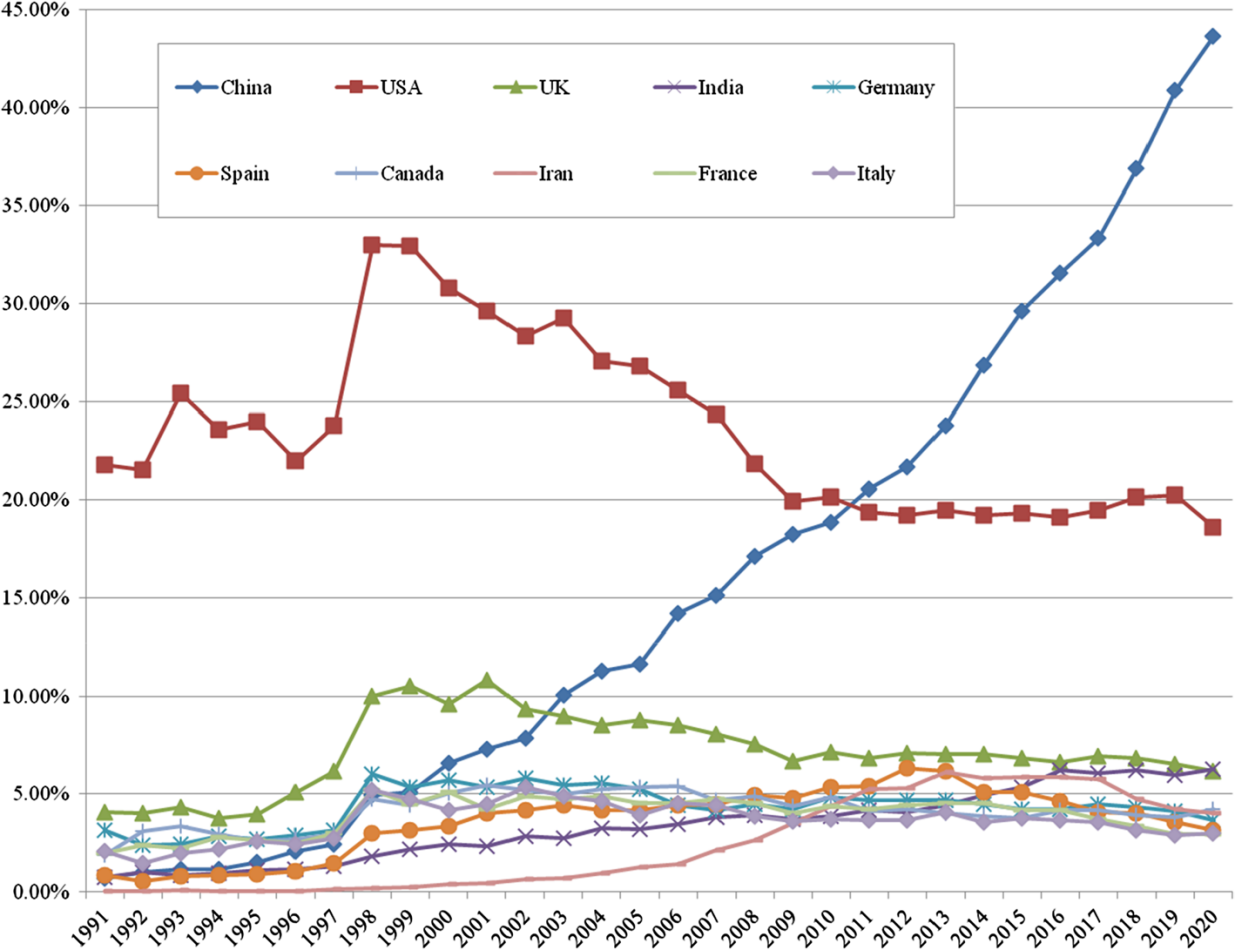
Annual world share of artificial intelligence articles for top ten countries, 1991–2020*. *Note* Analysis of WoS (SCI-E and SSCI) artificial intelligence articles published 1991–2020* (*N* = 464,373). Countries identified by author affiliations. 2020* = 24 May 2020

Our artificial intelligence publication dataset includes articles from 195 countries and territories, with more than 750,000 authors reported (without disambiguation). Yet, while researchers worldwide are involved in scientific publishing on artificial intelligence, a large proportion of the publication output is associated with a small group of leading countries. The top ten countries, by author affiliations, contributed to more than 70% of total worldwide artificial intelligence articles published in the period 1991–2020*. China and the US are the two most productive countries by the total number of artificial intelligence articles published, followed by the UK (Table 6). By world share of artificial intelligence articles, US-based authors were by far the leading producers in the first decade from 1991, rising to about one-third of all articles published by the end of the 1990s; there was then a decline in share in the next decade (Fig. 4). Since 2009, the US has maintained a share of about 20% of worldwide artificial intelligence article outputs. The trend is similar for the UK, with a rise to nearly 11% by the early 2000s, then declining towards the end of that decade but maintaining a consistent level of just under 7% throughout the 2010s. The greatest change in position is that of China, which has sharply increased its world share of artificial intelligence publications. By output volume, China passed the UK in 2003 and the US in 2011. Authors based in China are now the largest producers of artificial intelligence articles, contributing to just under 45% of the world’s output by 2020*. (In this paper, China refers to mainland China, Hong Kong, and Macau.)

**Table 6:** Publications and citations of artificial intelligence articles, top 10 countries, 1991–2020*

Measure	China	US	UK	India	Germany	Spain	Canada	Iran	France	Italy
Articles (× 1000)	118.0	99.4	32.8	21.5	20.4	19.6	19.3	18.2	18.0	16.5
All citations (× 1000)	1791.0	3385.9	941.1	327.0	534.4	376.5	538.6	250.8	482.2	356.5
Uncited articles (%)	21.5	11.9	11.6	19.5	12.8	12.0	12.4	14.8	13.0	11.8
Citations per article (mean)	15.2	34.1	28.7	15.2	26.2	19.2	27.9	13.8	26.9	21.6
H-index	294	549	306	159	242	178	227	124	229	184
H_m_	2.8	5.5	4.8	2.9	4.6	3.4	4.4	2.5	4.6	3.8
Top 10% cited (% country papers)	7.4	15.3	13.3	6.6	12.9	8.9	12.2	6.5	12.3	10.4
Top 1% cited (% country papers)	2.7	7.1	5.9	2.1	5.5	3.0	5.2	1.5	5.3	3.9

Analysis of WoS (SCI-E and SSCI) artificial intelligence articles published 1991–2020* (*N* = 464,373). See text for added details. 2020* = 24 May 2020. Countries identified by author affiliations

Of the other leading countries in the top ten, Canada, Germany, France, Italy and Spain each now contribute between 3.0 to 4.2% of the world total. India has seen steady growth in its share of world artificial intelligence articles, with its output very close to the UK by the end of the 2010s. Iran has also emerged as a noticeable producer of articles in artificial intelligence, although it reached its peak global share in 2013 and has since seen a declining global share (Fig. 4). Beyond the top ten, Taiwan, South Korea, Japan, Singapore, and Brazil are among the top twenty leading producers of artificial intelligence articles.

**Fig. 5:**
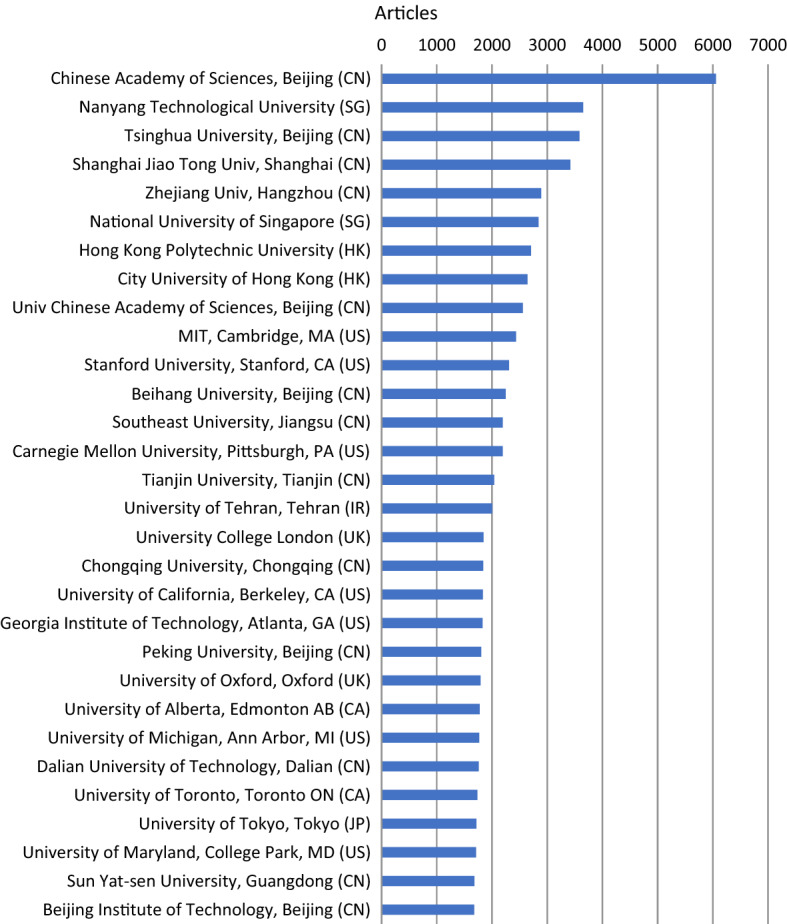
Top 30 organizations producing artificial intelligence articles, 1991–2020*. *Note* Analysis of WoS (SCI-E and SSCI) artificial intelligence articles published 1991–2020* (*N* = 464,373). Countries identified by author affiliations. Identified and aggregated by organization, city and country of author affiliations. 2020* = 24 May 2020

The dramatic rise of China in terms of the volume of artificial intelligence articles published is further evidenced by the significant presence of Chinese universities and institutes in the top thirty most productive organizations by artificial intelligence articles published from 1991 through to 2020* (Fig. 5). This analysis is based on the identification and aggregation by organization, city and country of author affiliations. Thirteen of the top 30 are universities or institutes based in mainland China, led by the Chinese Academy of Sciences (Beijing), Tsinghua University (Beijing), and Zhejiang University (Hangzhou), with a further two based in Hong Kong, led by Hong Kong Polytechnic University. Five of the top 30 productive organizations are in the US, including MIT, Stanford, and Carnegie Mellon University. Singapore, the UK, and Canada each have two organizations, including Nanyang Technological University (Singapore), University College London, and the University of Alberta (Edmonton). Iran and Japan each have one university among the top 30 most productive organizations, respectively the University of Tehran and the University of Tokyo.

### Citations to artificial intelligence articles

While volume of publication output is an important indicator of the scale of research activity, it is also vital to look at the quality of those outputs. While the drawbacks of using citation measures to assess publication quality are well recognized (Phelan 1999; van Raan 2019), citation data are widely used by scholars to assess the scientific influence of publications. To avoid limitations of using only one indicator, we calculate several citation-based indicators for artificial intelligence scientific articles for the top 10 countries (by author affiliations). We report total times cited and publication mean citations, noting that the first is related to the total number of publications while the second is susceptible to extreme citation values. Hence, we calculate composite citation-based indicators that consider both the quantity and quality of publications: the *H-* index, where *H* is the number of articles cited at least *H* times (Hirsch 2005); and the *H*_*m*_ = *H-*index/TN^0.4^ derived from the *H-*index and adjusted by the total number (TN) of articles (Molinari and Molinari 2008). Also computed is the share of worldwide highly cited articles for each country (Bornmann et al. 2012): we present measures of each country’s article outputs that are in the top 10% and top 1% of the most cited articles worldwide. Countries are identified by author affiliations.

Looking across these reported measures (Table 6), the US maintains the highest scientific influence in artificial intelligence: its total times cited, average times cited, *H* and *H*_*m*_ indices, and share of its output among the 10% and 1% worldwide most frequently-cited articles all rank first among the benchmark countries. The UK also performs strongly by these measures of scientific influence: for its artificial intelligence articles, measures for average citations, *H* and *H*_*m*_ indices, and share of output in the top 10% and top 1% of the most cited articles worldwide are high, coming in below the US but higher than the next group comprising of Germany, Canada, and France. In contrast, while China now leads by the absolute number of artificial intelligence articles produced over this nearly three-decade period, it lags in terms of its average article citation level, *H* and *H*_*m*_ indices, and share of output in the top 10% and top 1% of the most cited articles worldwide. China also has the highest number of uncited articles, at a rate that is almost twice as great as for the US and the UK. Two other Asian countries—India and Iran—are among the top ten countries by numbers of artificial intelligence articles published, although both also perform less strongly (and behind China) on most of the reported measures of scientific influence.

To observe dynamic changes in the scientific influences of the top countries (by volume of output) in the artificial intelligence field over successive time periods, we provide quinquennial calculations of the share of each country’s article output that is in the top 10% of the most cited articles worldwide (Table 7). In interpreting results, it should be noted that citation patterns are still formative in the early years after publication, although there is evidence of more reliability in citation impact measurement after a window of about three years (Adams 2005; Bornmann 2013). Over the long-run, the analysis confirms US leadership in the artificial intelligence field by this measure of scientific influence, ranking first among the compared countries in each five-year period. In the periods from 2000 to 2014, over 15% of US papers were in the top 10% most cited articles worldwide, although in the most recent 2015–2019 period, the US position diminished by more than two percentage points. Ranked second by this scientific influence measure, the UK broadly follows the US trend, rising in the share of its output in the top 10% most cited articles worldwide for the three quinquennial periods from 2000 to 2014, then dipping. However, in the 2015–2019 period, the gap between the US and the UK closed to just 0.4 percentage points. Three countries—Canada, Italy, and Iran—each saw increases in every five-year period in their share of outputs in the top 10% most cited articles worldwide, respectively ranking 3rd, 4th and 5th by this measure of scientific influence in the 2015–2019 period. Germany, which placed third by this measure in 2000–2004, saw its ranking fall to 6th place in 2015–2019. China’s share of outputs in the top 10% most cited articles worldwide grew noticeably in each of the three quinquennial periods from 2000 to 2014. In the most recent 2015–2019 period, there was no further growth (indeed a slight dip) in the share of China’s outputs in the top 10% most cited articles worldwide, although it might be noted that China’s performance on this metric was largely upheld notwithstanding a more than three-fold increase in annual article output in 2019 when compared with 2015. By share of outputs in the top 10% most cited artificial intelligence articles worldwide, China has narrowed the gap with the US, from 5.9 percentage points in the early 2000s to 1.5 percentage points towards the end of the 2010s. In this group of the leading 10 countries by article quantity, India demonstrated the weakest performance in the share of outputs in the top 10% most cited articles worldwide, although there was some modest improvement over the first three quinquennials of the twenty-year period (Table 7).

**Table 7:** Country share of top 10% of the most cited artificial intelligence articles worldwide, 2000–2019

	2000–2004	2005–2009	2010–2014	2015–2019
All articles, worldwide (× 1000)	36.0	58.9	102.8	195.8
In worldwide top 10% most-cited	%	%	%	%
US	15.2	15.3	15.4	13.0
UK	12.4	13.4	14.0	12.6
Canada	9.8	12.2	12.9	12.2
Italy	8.0	9.2	10.2	11.8
Iran	4.0	6.0	7.8	11.7
Germany	10.6	14.0	14.5	11.6
China	9.3	10.2	11.6	11.5
France	10.5	11.8	11.6	10.9
Spain	7.1	7.8	9.2	9.5
India	7.4	8.0	8.8	8.5

Analysis of WoS (SCI-E and SSCI) artificial intelligence articles published 1991–2019 (*N* = 393,439). Top ten countries by output of articles. Countries identified by author affiliations

### Co-author collaboration across countries

Researchers increasingly collaborate in teams within and across institutional and national boundaries in order to leverage knowledge, disciplinary and interdisciplinary capabilities, scientific infrastructure, reputational benefits, and other resources (Glänzel and Schubert 2004; Bozeman and Youtie 2017; Chen et al. 2019). Consistent with this broad trend, the co-authorship of scientific publications is predominant in the artificial intelligence research domain. In our WoS dataset of over 464,000 artificial intelligence articles (1991–2020*), just 8.6% are single authored, nearly a half (48.9%) have two or three authors, more than one third (34.9%) have four-to-six authors, and 7.7% have seven or more authors. Many of these co-authorships are multi-institutional. More than one-half (53.8%) of artificial intelligence articles involve authors with two or more organizational affiliations.

We also find that co-authorships for artificial intelligence research are frequently international, although there are differences among the leading producers of scientific articles in this domain. For the period 1991–2019, about 41% of US artificial intelligence articles are internationally co-authored, most noticeably with China (accounting for 14% of all US artificial intelligence papers), followed by the UK (4%) and Canada (3%) (Table 8). International co-authorship is noticeably lower for China, where about 31% of artificial intelligence articles are internationally co-authored, with the USA contributing to over one-tenth of Chinese publications in the field. The percent of internationally co-authored publications for Iran is just below the Chinese level, at about 30%, while for India it is 23%—the lowest among the top ten publishing countries. The UK has the highest level of international co-authorship, with nearly three-fifths of its artificial intelligence papers being international co-authored. The UK’s international partners are led by China (15% of UK papers) and the US (12%), followed by Germany (6%). Canada, Germany and France also have a high international co-authorship rate (all over 50%), with the US, the UK and China as their leading collaborators.

**Table 8:** International co-authoring for top 10 artificial intelligence publishing countries, 1991–2020*

	International co-authored articles			Leading co-authoring countries					
	× 1000	Percent	Countries	First		Second		Third	
				Country	Percent	Country	Percent	Country	Percent
USA	40.9	41.1	164	China	14.0	UK	4.1	Canada	3.3
China	36.3	30.7	127	US	11.8	UK	4.2	Australia	3.7
UK	18.9	57.7	151	China	15.2	US	12.4	Germany	6.3
Germany	11.0	54.1	142	US	15.2	UK	10.1	China	5.8
Canada	10.8	56.0	130	US	16.9	China	16.6	UK	4.5
France	9.7	53.9	138	US	11.7	UK	6.8	China	6.3
Spain	8.2	41.7	129	US	7.9	UK	7.7	France	4.5
Italy	7.6	46.2	133	US	12.1	UK	9.1	France	6.6
Iran	5.4	29.7	96	US	5.8	Canada	4.2	Malaysia	3.7
India	4.9	22.9	112	US	6.4	China	3.3	South Korea	2.1

Analysis of WoS (SCI-E and SSCI) artificial intelligence articles published 1991–2020* (*N* = 464,373). Top ten countries by output of articles. 2020* = 24 May 2020. Countries identified by author affiliations. Percent refers to portion of article output of each top ten country

**Fig. 6:**
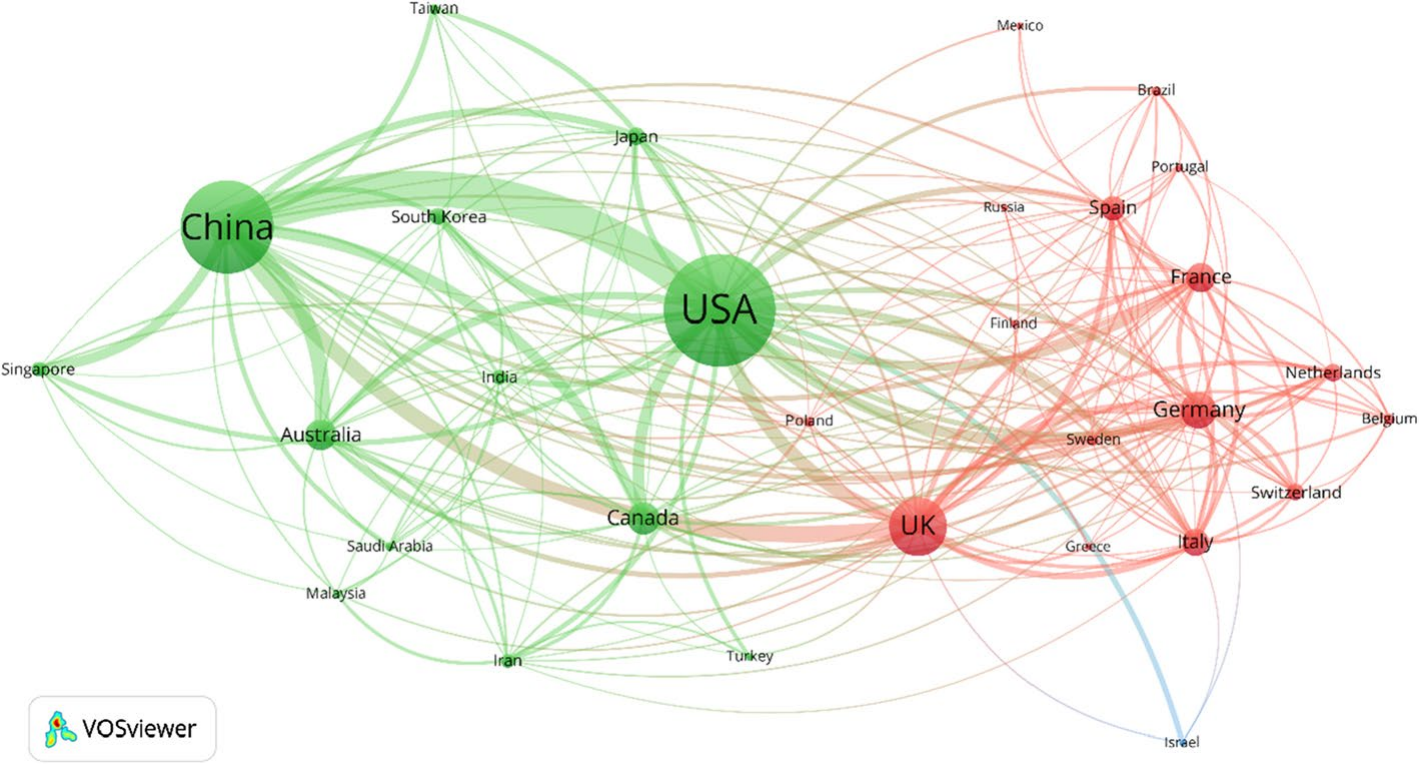
Artificial intelligence co-author collaboration networks, top 30 countries. *Note* Analysis of WoS (SCI-E and SSCI) artificial intelligence articles published 1991–2020* (*N* = 464,373). 2020* = 24 May 2020. Visualization using VOSviewer, nodes represent countries (identified by author affiliations) and linkages represent co-authorship relationships between countries

Patterns of collaboration between countries in artificial intelligence scientific research are further revealed through an international co-authorship network map for the top 30 countries (by volume of output, 1991–2020*) (Fig. 6). The US, as the leading partner of most other top countries, plays a dominant role in artificial intelligence transnational co-authorship linkages. China and the UK also serve as next tier hubs in transnational networks. China and the US are the most linked pair of countries, by volume of co-authored articles. With China and the US as dual hubs, there is an Asia–Pacific cluster, also involving Australia, Singapore, Canada, Japan and Taiwan. A clustered European network is also evident, with the UK, Germany, and France as key nodes.

### Research sponsors of artificial intelligence

Further insights into the landscape of artificial intelligence research can be gleaned by investigating research sponsors. Research sponsors are influential in guiding what research is supported, who gets support, and how they are supported. Funding acknowledgement information is first available in the WoS from mid-2008. Research in papers that do not report funding acknowledgements may have been aided through institutional resources rather than specific grant award. However, if a particular grant or funding source was received, it is likely to be reported, as funding sponsors and journals now typically require that recipients acknowledge funding support. The organizational name of the funding sponsor and often the specific grant program and award number is reported, although not the amount of funding. Individual papers may acknowledge more than one funding sponsor from one or more countries, depending on their co-authorship arrangements. Since the same funding sponsor may be reported by authors and journals in varied ways, we applied a text matching, cleaning and manual review process to our WoS dataset to develop a robust and validated set of sponsor names (Wang and Shapira 2011).

Beginning from the subsequent first full year of information on funding in the WoS, we find that 66.9% of 339,347 artificial intelligence articles published during the period 2009–2020* report funding acknowledgements information. Among the leading countries by output of artificial intelligence articles, China has the highest share (88.6%) of articles that report funding acknowledgements. For the US and the UK, respectively 72.5% and 69.8% of articles report funding acknowledgements. Just over 70% of articles by authors with affiliations in Germany and Canada report funding acknowledgements. At the lowest end are India and Iran, where respectively 30.6% and 21.6% of articles report funding acknowledgements.

**Fig. 7:**
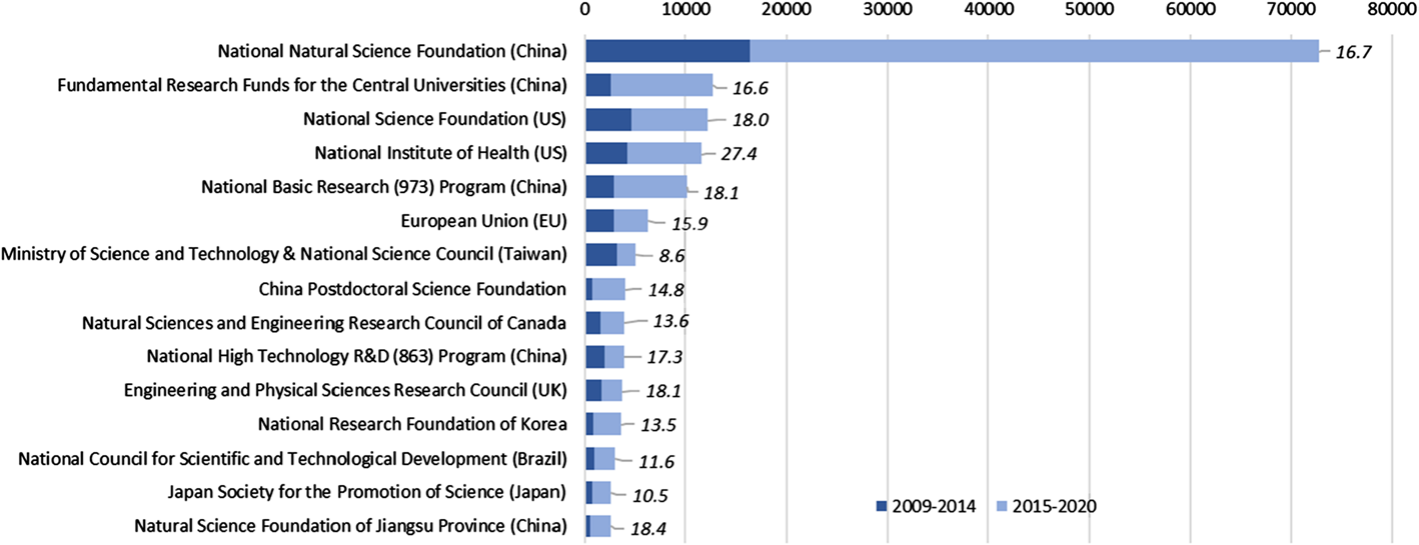
Top 15 funding sponsors acknowledged in artificial intelligence articles, 2009–2020*. *Note* Analysis of WoS (SCI-E and SSCI) articles, 2009–2020*, AI search (*N* = 339,347). 2020* = 24 May 2020. Data label to right of each bar is average citations through to 2020* for articles published in 2016 and 2017 acknowledging that funding sponsor

A relatively small group of sponsors are prominent (by number of funding acknowledgements reported) in their support of funded research in the artificial intelligence research domain. The top 30 sponsors are acknowledged in more than four-fifths (82.8%) of articles that report funding acknowledgements. All are public research support bodies or agencies associated with government. We focus on the top 15 research sponsors, which are acknowledged in more than 158,000 artificial intelligence articles published between 2009–2020*—equivalent to 69.6% of all papers in this period that report funding acknowledgements. Overall, China has five sponsors among these top 15 funders of artificial intelligence research, the US has three, two are in Europe, and Taiwan, Canada, South Korea, Brazil and Japan each have one (Fig. 7). The growth of the National Natural Science Foundation (NNSF) of China as a funder of artificial intelligence research is particularly noticeable. By 2014, NNSF was already the world’s largest sponsor of research in this domain, as reported by funding acknowledgements; by 2020*, it had moved yet further ahead. Between 2015 and 2020*, more than 56,000 artificial intelligence articles acknowledged NNSF support—a sum that was greater than the number of papers supported during this period from the other 14 sponsors combined. Other leading funding agencies outside of China also increased the number of artificial intelligence papers supported, but not at the same rate. When the first period (2009–2014) is compared with the second period (2015–2020*), artificial intelligence articles acknowledging NNSF support increased by 242%. For the two largest US sponsors, the National Science Foundation (NSF) and the National Institutes of Health (NIH), the equivalent growth rate was 62% and 72% respectively, while for the UK Engineering and Physical Sciences Research Council (EPSRC), the growth rate was 29%. Other Asian funding sponsors saw higher growth rates in funding acknowledgements between these two time periods, for example South Korea’s National Research Foundation increased by 204%, but from a much lower base than for NNSF.

While NNSF and other sponsors in China and elsewhere have increased the quantity of research outputs supported in the artificial intelligence domain, we also probe the quality of recent publications underwritten by the top 15 research sponsors. Given the rapid growth of research outputs, we sought an appropriate time window that would capture relatively recent publications yet allow sufficient time for citation patterns to emerge. As noted in the earlier discussion on citations to artificial intelligence articles, a 3-year citation window can be viewed as appropriate. We thus focus on articles published in 2016–2017, which (given our data end point of 24 May 2020) provides an average article age of 3.3 years. In this period, almost 15,000 articles published in 2016 and 2017 acknowledge NNSF funding support, with just over 2,800 articles acknowledging support from Fundamental Research Funds from the Central Universities (FRFCU) of China. Over 2100 artificial intelligence articles published in 2016 and 2017 acknowledge funding support from each of the US NSF and NIH, with about 1000 acknowledging support from European Union sources. The other non-Chinese research bodies are acknowledged in the range of 500 to just under 800 articles published in 2016 and 2017. In the subsequent three-year period through to 2020*, publications funded by the US NIH garner the highest average citations with 27.4 per article; publications supported by the UK EPSRC attract an average of 18.1 citations per article, while for the US NSF the average is 18.0 citations per article (Fig. 7). Articles supported by China’s NNSF and FRFCU attract fewer cites on average, at 16.7 and 16.6 citations per article. Nonetheless, papers that acknowledge NNSF and FRFCU funding are cited, on average and in our three-year time window, at higher rates than for publications supported by the European Union and sponsors in Canada, South Korea, Japan and Brazil. Additionally, for articles supported by China’s 973 Program and by the Jiangsu Province National Science Foundation, average citation levels are comparable to those of EPSRC and the US NSF. This analysis does not take into account field differences in citation patterns and distributions around the mean for citations. Nor does it adjust for different patterns in citations within countries. However, it does suggest that the massive push to expand support for artificial intelligence scientific research in China has not necessarily come at the expense of quality, at least as measured by average citations to relatively recent papers.

### Scientific disciplines of artificial intelligence

The inherently multidisciplinary nature of artificial intelligence (Sombattheera et al. 2012) is clearly evident by the range of WoS subject categories involved in artificial intelligence publications. Each journal in which a paper is published is classified by the WoS into one or more of over 250 granular subject categories (including multidisciplinary sciences if a journal covers more than six subject categories). Some 243 WoS subject categories are represented by the articles captured in our data set. However, a smaller number of subject categories encompasses a majority of these articles. The top 15 subject categories together cover 69.4% of all WoS artificial intelligence articles in the period 1991–2020* (Table 9). The leading subject category is “computer science, artificial intelligence”, covering about 40% of artificial intelligence articles in the most recent period of 2011–2020*, followed by “engineering, electrical & electronic” and “computer science, information systems” with 23% and 10% respectively. There is also the suggestion of a diffusion of artificial intelligence concepts and methods into other subject categories. The core topic of “computer science, artificial intelligence” dropped down in its share of artificial intelligence articles by about 11 percentage points between 1991–2000 and 2011–2020*, even though increasing in absolute numbers of publications, as other subject categories grew over these periods, including “telecommunications”, “computer science, information systems” and other non-computer science related categories.

**Table 9:** Top 15 WoS subject categories of artificial intelligence articles, 1991–2020*

	Publication year			
	Total	1991–2000	2001–2010	2011–2020*
Articles (× 1000)	464.4	51.8	104.5	308.1
Web of science category	Percentage of total articles (%)			
Computer science, artificial intelligence	43.8	51.1	50.7	40.2
Engineering, electrical and electronic	23.3	26.0	24.0	22.6
Computer science, information systems	8.9	6.3	6.9	10.0
Computer science, interdisciplinary applications	7.6	5.4	7.4	8.1
Automation and control systems	6.5	6.7	7.5	6.1
Computer science, theory and methods	6.2	9.2	7.1	5.3
Neurosciences	4.8	7.0	5.6	4.1
Operations research and management science	4.6	4.3	6.0	4.1
Telecommunications	3.5	0.9	1.0	4.8
Computer science, software engineering	3.4	4.0	3.5	3.3
Engineering, multidisciplinary	3.2	2.8	2.6	3.4
Instruments and instrumentation	3.1	3.3	2.6	3.2
Computer science, cybernetics	2.6	4.8	3.6	1.9
Mathematics, applied	2.4	2.4	3.1	2.2
Chemistry, analytical	2.3	2.8	2.4	2.2
Computer science related categories	55.3	63.3	59.4	52.5
Non-computer science related categories	76.1	73.5	76.2	76.5

Analysis of WoS (SCI-E and SSCI) artificial intelligence articles published 1991–2020* (*N* = 464,373). Total of 243 subject categories. Computer Science related categories include “Computer Science, Artificial Intelligence”, “Computer Science, Information Systems”, “Computer Science, Interdisciplinary Applications”, “Computer Science, Theory & Methods”, “Computer Science, Software Engineering”, “Computer Science, Cybernetics”, “Computer Science, Hardware & Architecture” and “Robotics”

**Fig. 8:**
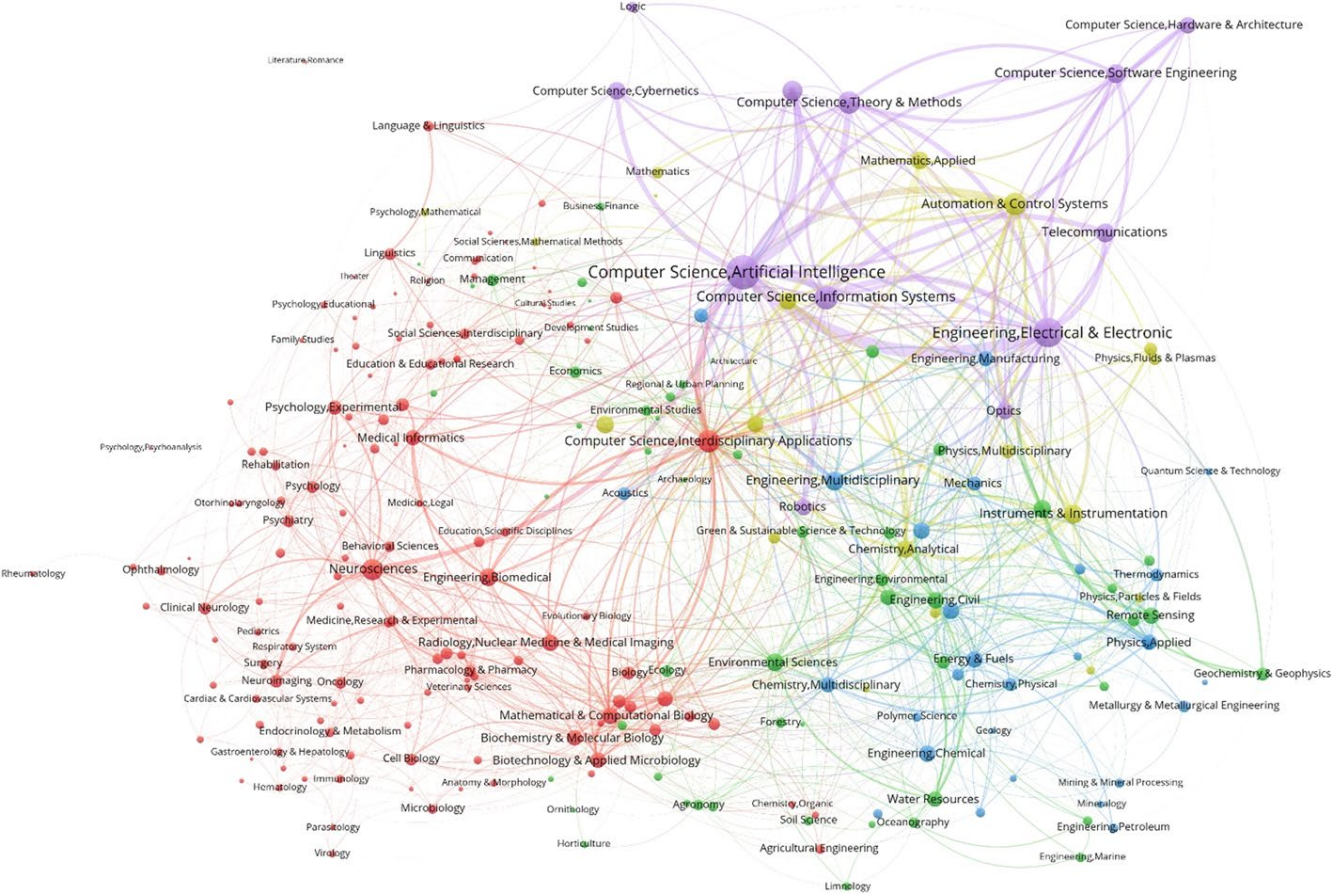
Profile of artificial intelligence research by clusters and subject categories. *Note* Analysis of WoS (SCI-E and SSCI) artificial intelligence articles published 1991–2020* (*N* = 464,373). 2020* = 24 May 2020. Total of 243 WoS subject categories, visualization using VOSviewer, nodes represent subject categories and linkages represent co-occurrence relationships among them

To further explore the distribution of subject categories and the linkages among them, we constructed a co-occurrence network map which we visualize using VOSviewer (Fig. 8). We can observe five clusters in this map. A first (purple) cluster involves computer science and engineering related categories including “computer science, artificial intelligence”, “engineering, electrical & electronic”, “computer science, theory & methods”, “telecommunications” and “cybernetics”. A second (red) cluster involves “computer science, interdisciplinary applications”, “neurosciences” and multiple medical and biology related categories. A third (yellow) cluster involves “automation & control systems”, “instruments & instrumentation” and linked categories of mathematics, chemistry and physics. A fourth (blue) cluster includes categories related to engineering, manufacturing and materials science. Finally, a fifth (green) cluster includes “environmental sciences”, “remote sensing”, “engineering environmental”, “engineering, civil” and “water resources” and social sciences such as “management”, “business, finance” and “economics”. This co-occurrence visualization of subject categories shows a wide spread of artificial intelligence publications across macro-disciplines and subject categories. The map also highlights the emergence of multi-disciplinary assemblages of scientific activities engaged not only in the development of artificial intelligence concepts and hardware and control systems but also and in artificial intelligence applications especially in industrial, materials, environmental, and life science areas.

## Discussion

As explained in the paper, we develop and apply a new search approach to map the global landscape of artificial intelligence scientific research. We analyzed articles published in the artificial intelligence domain, examining outputs over time, by countries and organizations, citations, transnational co-author collaborations, research sponsorship and the distribution of scientific disciplines.

We find a sustained growth in artificial intelligence scientific research outputs over the last three decades, with a significant acceleration in the last five years (Since 2016). The US and UK were early movers in artificial intelligence scientific research, and their outputs continue to grow. However, the largest quickening of output is seen in China, which now leads all other countries by volume of papers produced in artificial intelligence. The increasing level of scientific capability that China is building, and which can be observed in research publications, is likely to have spillover effects, through knowledge and human capital development, for its governmental and industrial efforts in artificial intelligence. Although China’s scientific influence, as measured by citations to published articles, still trails the US and the UK, there has been a clear rise in citation quality of Chinese papers in artificial intelligence to levels that in recent years are higher than for Canada and some other European countries.

Yet, notwithstanding that individual countries have sought to promote their capabilities in artificial intelligence research, we also find widespread international co-author collaboration in this field, with the US, China, and the UK among the hubs for international collaboration networks. The growth of scientific research in artificial intelligence is primarily supported through public funding, as we highlighted by identifying the leading research sponsors acknowledged in published articles. Additionally, while scientific research in artificial intelligence clusters in computer science and information technology areas, we see that artificial intelligence concepts and methods are spreading to other field including those related to automation, biomedicine, materials, and manufacturing.

## Conclusions

In this final section, we provide concluding comments, consider limitations, and highlight further research opportunities.

The paper has put forward a systematic method for constructing a bibliometric definition for the field of artificial intelligence. We explained the stages in our process in detail, making it possible for others to replicate the approach. The resulting search strategy was evaluated by comparing its search records and search terms with the counterparts of three other search strategies used in previous bibliometric analyses of artificial intelligence. This comparison suggests that these extant search strategies for artificial intelligence are either too narrow or too broad. This benchmark assessment indicated that our search strategy offers an appropriate and justified balance between recall and precision.

We position the artificial intelligence search strategy defined in this paper as a public tool. It is available for other researchers to use and refine. The search approach can also be employed by technology managers, research funders, policy analysts, and others interested in research publication activity in the artificial intelligence domain. The steps involved in applying it to the Web of Science are straightforward (directly using the search strategy as defined in Table 5 involving search keywords and a subject category). The search is readily adaptable for use in other bibliometric databases, such as Scopus or in patent databases. We note that there may be a need to adjust how the search strategy is inputted. For example, to use the search strategy in Scopus, for the equivalent of the “artificial intelligence” subject category, the All Science Journal Classification Code (ASJC) for “artificial intelligence” can be applied to develop an appropriate journal list. Additionally, for patent databases (such as Derwent Innovations, PATSTAT or PatentSight), the International Patent Classification (IPC) or Cooperative Patent Classification (CPC) codes can be used to refine the keyword-based search.

There are limitations that should be kept in mind when interpreting or applying our approach. The limitations of the Web of Science in terms of global journal coverage, subject category representation, and over-representation of English language publications are well-documented (Mongeon and Paul-Hus 2016). Our focus is on artificial intelligence scientific research outputs as published in articles in journals in the WoS SCI-Expanded and SSCI databases; while we contend that this is an appropriate source, especially to indicate trends and patterns, we note that we do not analyze non-journal preprints, non-journal conference papers, books, or other databases. We further note that artificial intelligence is an evolving domain and will surely give rise to search terms that we do not currently capture. Moreover, while we maintain that the “Hit Ratio” provides a rational way to assess the relevance of candidate terms in a specific field, there is no agreed standard for its threshold values. The inclusion, review, and exclusion values we use are based on judgement and iterative trial and error. Other researchers can update the search strategy by adding new artificial intelligence terms or journals using the bibliometric search process that we have described, and they can also apply variations to Hit Ratios to see if recall and precision in future searches can be improved.

The construction and application of our bibliometric definition to track the profile of scientific developments in artificial intelligence is a contribution to what must be an ongoing domain of study. Artificial intelligence is developing as one of the key platform technologies of our generation, accompanied by both promise and concern about its design and implementation. In our own work, we intend to apply the search approach to analyze patents; this will assist in mapping inventions, applications, and corporate activities that use artificial intelligence concepts and methods. We are engaging in work to explore emerging innovation ecosystems at regional, national, and international levels and in how artificial intelligence is being applied in laboratory sciences. There are many other opportunities for future studies of artificial intelligence research and innovation. We trust that the bibliometric search approach presented in this study can help to inform these studies.
